# The Impact of Age on Outcomes in Seizure Hospitalizations—Analysis of a National Sample

**DOI:** 10.3390/neurolint17030039

**Published:** 2025-03-04

**Authors:** Anudeep Surendranath, Saurabh Singhal, Rahul Khanna, Subhendu Rath, Temenuzhka Mihaylova

**Affiliations:** 1 Neurology, CHI St. Vincent—Hot Springs, 1662 Higdon Ferry Rd. Suite 100, Hot Springs, AR 71913, USA; 2 Neurology, Well Smart Health, Neurology Clinic, 1270 Attakapas Suite 401, Opelousas, LA 70570, USA; saurabhsinghal110@gmail.com; 3 Neurology, Neurology and Sleep Clinic, PLC, 1225 S Gear Ave. #153, West Burlington, IA 52655, USA; rahulkhanna.doctor@gmail.com; 4 Neurology, University of Kentucky, 740 S. Limestone First Floor, Wing C, Room B101, Lexington, KY 40536, USA; subhendu.rath@uky.edu; 5 Neurology, University of Michigan, F593 UH South, SPC 5223, 1500 East Medical Center, Ann Arbor, MI 48109, USA; tmihaylo@med.umich.edu

**Keywords:** seizures, age factors, hospital mortality, length of stay, healthcare costs, comorbidities

## Abstract

**Highlights:**

**What are the main findings?**

- Older adults hospitalized with seizures have over twice the odds of in-hospital mortality compared with younger adults, even after adjusting for confounders.
- Seizure-related hospitalizations in older adults are associated with longer hospital stays and higher healthcare costs.
- Older adults with seizures exhibit distinct demographics and a greater burden of comorbidities.

**What is the implication of the main finding?**

- Tailored, age-specific management strategies are needed to improve outcomes for older adults hospitalized with seizures.
- National data highlight the significant healthcare burden of seizures in the aging population.
- Optimizing healthcare resource utilization and improving clinical care strategies for older adults with seizures is essential to reducing mortality and hospitalization costs.

**Abstract:**

**Objective:** Seizures are a critical public health issue, with incidence rising significantly after age 50. Using this inflection point, we divided patients into two age groups to examine the impact of age on patient characteristics and hospitalization outcomes for seizures. **Methods:** Using the 2021 National Inpatient Sample (NIS), a nationally representative database, we conducted a retrospective cohort analysis of adult patients aged ≥18 years admitted with a principal diagnosis of seizures. Patients were divided into two age groups: 18–49 and ≥50 years. Outcomes included in-hospital mortality, length of stay, and hospital charges. Multivariate logistic and linear regression models adjusted for confounders were employed to assess the association between age and outcomes. **Results:** The cohort included 211,055 patients, with 59% aged ≥50 years. Older patients were more likely to have Medicare coverage (66% vs. 16%, *p* < 0.01), to reside in the south (41% vs. 38%, *p* < 0.01), and to have a higher proportion of White individuals (62% vs. 54%, *p* < 0.01). Younger patients were more likely to be Hispanic (15% vs. 9%, *p* < 0.01), admitted to urban hospitals (96% vs. 94%, *p* < 0.01), and treated at teaching hospitals (84% vs. 79%, *p* < 0.01). After adjusting for confounders, older adults had over twice the odds of in-hospital mortality compared with younger patients (adjusted OR 2.17; 95% CI, 1.61–2.92; *p* < 0.01). They also experienced longer hospital stays (mean difference 0.7 days; 95% CI, 0.54–0.92; *p* < 0.01) and higher hospital charges (mean increase USD 4322; 95% CI, USD 1914–6731; *p* < 0.01). **Significance:** Age is an independent predictor of in-hospital mortality, longer hospitalizations, and higher costs in seizure-related admissions. These findings underscore the need for age-specific management strategies to improve outcomes and optimize healthcare resource utilization for older adults with seizures.

## 1. Introduction

Seizures represent a critical public health issue and are among the most common reasons for inpatient neurology consultations [1]. Affecting approximately 1.1% of the population, the incidence of seizures demonstrates a bimodal distribution, with a notable increase after the age of 50 [2,3,4,5,6]. Although no universally accepted threshold defines “late-onset” seizures, this demographic shift highlights the growing prevalence among older adults. The aging population in the United States and globally underscores the importance of understanding how age impacts patient characteristics and outcomes in seizure-related hospitalizations. The rising prevalence of late-onset seizures, often linked to cerebrovascular disease and neurodegenerative changes, highlights the need for improved clinical awareness and tailored management strategies. This demographic shift brings new challenges and opportunities for optimizing care delivery for older adults.

Epilepsy among older adults has been identified as a cause of global health concern. In regions with increasing elderly populations, older adults significantly contribute to the growing burden of epilepsy-related hospitalizations. This is related to associated comorbidities and higher risks of potential complications compared with younger patients [7]. Studies indicate that cerebrovascular and degenerative changes in the aging brain heighten the susceptibility to seizures, along with presenting diagnostic challenges. Notably, seizures in the elderly often present atypically, frequently mimicking other neurologic disorders such as transient ischemic attacks, which may delay appropriate treatment [8]. The economic implications of epilepsy in aging populations are significant. Older adults with epilepsy are at a higher risk for readmissions. Chronic concurrent disorders such as strokes, dementia, and transient metabolic disturbances can complicate their management. Additionally, the increasing use of polypharmacy in this demographic elevates the risk of adverse drug interactions with inferior outcomes [9]. These challenges require an in-depth understanding of various age-specific processes that influence epilepsy-related hospitalizations and necessitate the utilization of targeted interventions.

To address this knowledge gap, we conducted a retrospective cohort study utilizing data from the 2021 National Inpatient Sample (NIS), curated by the Agency for Healthcare Research and Quality (AHRQ). This analysis aimed to explore the influence of age on patient demographics, clinical characteristics, and hospitalization outcomes in individuals admitted with a principal diagnosis of seizures. By comparing younger (aged 18–49) and older (aged 50 and above) patients, we sought to elucidate age-related trends and their implications for healthcare delivery and resource utilization.

## 2. Methods

### 2.1. Study Design and Database Description

This retrospective cohort study was conducted using data from the 2021 National Inpatient Sample (NIS), which is part of the Healthcare Cost and Utilization Project (HCUP). Curated by the Agency for Healthcare Research and Quality (AHRQ), the NIS provides a comprehensive overview of hospital discharges in the United States, covering 97% of the population and 96% of hospital discharges. The database’s stratified sampling design ensures representativeness by including hospitals based on ownership, size, teaching status, and geographic location. The patient-level data are de-identified to maintain confidentiality, while state and hospital identifiers are excluded.

The NIS dataset includes a 20% stratified sample of all U.S. hospital discharges. This large sample size not only enhances the ability to detect differences in outcomes across various subgroups but also ensures reliable extrapolation to national estimates, providing a robust foundation for evaluating age-related trends in seizure hospitalizations. Additionally, the inclusion of stratified sampling weights allows researchers to generate nationally representative estimates, making it an invaluable resource for studying population-level trends and healthcare outcomes. Since the data are de-identified and publicly available, Institutional Review Board (IRB) approval and informed consent were not required for this study.

### 2.2. Study Population

We included all adults aged 18 years and older who were admitted with a principal diagnosis of seizures. Patients were identified through ICD-10 codes (provided in the Supplementary Table S1), and two age groups were established to analyze the age-related impacts: one for individuals aged 18–49 and another for those aged 50 and older. We then compared patient characteristics and outcomes between these two groups to examine age-related trends.

### 2.3. Outcomes of Interest

The primary outcome was in-hospital mortality. Secondary outcomes included the length of stay (LOS) and total hospital charges. These outcomes were chosen for their relevance to assessing both the clinical and economic impacts of seizure-related hospitalizations. By focusing on these outcomes, this study aimed to provide insights into both the patient-level and system-level implications of seizure management.

### 2.4. Statistical Analysis

Data analysis was performed using Stata version 18. Continuous variables were expressed as means with standard deviations, while categorical variables were presented as frequencies and percentages. T-tests were used to calculate *p*-values for continuous variables, while Fisher’s exact test was applied to binary and categorical variables. For multivariate analyses, logistic regression was employed to assess the association between age groups and in-hospital mortality, with results presented as odds ratios (ORs) and 95% confidence intervals (CIs). Linear regression was used for continuous outcomes (LOS and hospital charges), reporting mean differences and 95% CIs. Statistical significance was defined as a two-tailed *p*-value < 0.05.

## 3. Results

### 3.1. Patient Demographics and Hospital Characteristics

The NIS dataset included 33.3 million observations, among which 211,055 were adult patients admitted with a principal diagnosis of seizures. Within this subset, 123,885 (59%) were aged 50 or older. Table 1 presents a comparison of patient and hospital characteristics for the two age groups. The mean age of patients in the younger group (aged 18–49) was 33.63 years, while the mean age of those 50 and older was 67.34 years. A greater proportion of women were represented in the older age group (49% vs. 46%, *p* < 0.01). Differences in racial distribution were observed, with a higher percentage of White patients in the older age group (62% vs. 54%, *p* < 0.01) and a higher percentage of Hispanic patients in the younger cohort (15% vs. 9%, *p* < 0.01). Medicare coverage was substantially higher in the older age group (66% vs. 16%, *p* < 0.01), while Medicaid, private insurance, and self-pay options were more common among younger patients. Geographically, older patients were more prevalent in the south (41% vs. 38%, *p* < 0.01), and younger patients were slightly more likely to be admitted to urban hospitals and teaching facilities (*p* < 0.01).

### 3.2. Distribution of Clinical Characteristics

The incidence of status epilepticus during the index hospitalization did not differ significantly between the two age groups (*p* = 0.07). As anticipated, younger patients had lower Charlson Comorbidity Index scores, while older patients showed higher scores, a difference that was statistically significant. For other comorbidities, which could reflect prior medical history rather than conditions present during the index hospitalization, the prevalence of ischemic stroke, nontraumatic intracerebral hemorrhage, dementia, and brain neoplasms was notably higher among older patients. However, there was no statistically significant difference in the proportion of patients with traumatic brain injury (TBI) across the two age groups. Table 2 presents a comparison of the relevant clinical characteristics for the two age groups. Treatments and tests results are important clinical characteristics that were not analyzed in our study due to a lack of data.

### 3.3. Primary Outcome

Out of the 211,055 adult inpatients admitted with a principal diagnosis of seizures, 2805 (1.33%) died during their hospitalization. The unadjusted odds ratio (OR) for mortality in the older age group was 3.73 (95% confidence interval [CI], 2.95–4.73; *p* < 0.01). After adjusting for confounders—including gender, race, median income level, hospital region, Charlson Comorbidity Index, urban location, teaching hospital status, and relevant comorbidities such as status epilepticus, ischemic stroke, nontraumatic intracerebral hemorrhage, TBI, dementia, and brain neoplasms—the adjusted OR for mortality in the older group was 2.17 (95% CI, 1.61–2.92; *p* < 0.01). This increased mortality risk persisted even after adjusting for these comorbidities, highlighting age as an independent predictor of in-hospital death. These findings underscore the substantial clinical impact of seizures in older adults. These results are summarized in Table 3. Similar results were obtained when adjusting only for factors with *p* < 0.2 on univariate analysis, further validating the observed association between age and increased mortality risk.

### 3.4. Secondary Outcomes

The mean length of stay for patients admitted with a principal diagnosis of seizures was 4.5 days. In the adjusted analysis, patients aged 50 and older had an average stay that was 0.7 days longer than that of younger patients (95% CI, 0.54–0.92 days; *p* < 0.01). Hospital charges for seizure admissions averaged USD 59,805, with adjusted charges showing an increase of USD 4322 for the older group (95% CI, USD 1914–6731; *p* < 0.01) compared with their younger counterparts. These secondary outcome results, including length of stay and hospital charges, are detailed in Table 4, highlighting the substantial impact of age on resource utilization in seizure-related hospitalizations.

## 4. Discussion

The global lifetime age-standardized prevalence of epilepsy is 621.5 per 100,000 population [1]. In the United States, approximately 1.1% of adults have active epilepsy, amounting to about 2.9 million individuals [2]. The incidence of epilepsy demonstrates a bimodal distribution, with cases peaking in individuals under five years of age and increasing later in life [3,4]. This incidence trend is especially pronounced after the age of 50 [5,6]. Consequently, we studied the distribution of hospital, patient, and clinical characteristics and outcomes among patients admitted with a principal diagnosis of seizures before and after this age threshold.

Our analysis of 211,055 adult patients admitted with a principal diagnosis of seizures highlights notable age-related differences in patient demographics and hospital characteristics. Older adults (aged 50 years or older) accounted for 59% of the cohort and were more likely to have Medicare coverage (66% vs. 16%, *p* < 0.01) and a higher proportion of White individuals (62% vs. 54%, *p* < 0.01). Conversely, younger patients (aged 18–49) were more likely to be Hispanic (15% vs. 9%, *p* < 0.01), admitted to urban hospitals (96% vs. 94%, *p* < 0.01), and treated at teaching hospitals (84% vs. 79%, *p* < 0.01). Geographically, older adults were slightly more concentrated in the south (41% vs. 38%, *p* < 0.01), while the Charlson Comorbidity Index scores were higher in older patients, with 34.6% scoring ≥3 compared with only 7.0% of younger patients (*p* < 0.01).

In terms of outcomes, older adults exhibited worse primary and secondary outcomes compared with their younger counterparts. The adjusted odds of in-hospital mortality for older adults were more than double (adjusted OR 2.17; 95% CI, 1.61–2.92; *p* < 0.01). The mean length of hospital stay was significantly longer for older adults, with an average difference of 0.7 days (95% CI, 0.54–0.92; *p* < 0.01). Additionally, hospital charges were significantly higher for older adults, with an adjusted mean increase of USD 4322 (95% CI, USD 1914–6731; *p* < 0.01). These differences in outcomes persisted despite adjusting for confounders, including comorbidities, gender, race, and hospital characteristics.

Stroke is frequently identified as a leading cause of late-onset epilepsy [6,10,11]. Other common causes include metabolic disturbances, medication effects, dementia, and intracranial neoplasms [12,13]. In our cohort, the prevalence of ischemic stroke, nontraumatic intracerebral hemorrhage, dementia, and brain neoplasms was notably higher among older patients, consistent with the literature. However, there was no significant difference between age groups regarding the prevalence of traumatic brain injury (TBI).

Acute symptomatic seizures are a common occurrence in older adults, frequently precipitated by acute cerebrovascular events, metabolic disturbances, or systemic insults [14,15]. They account for a significant proportion of first-time seizures in this population and have an incidence that rises sharply with age, peaking after 65 years [14]. These seizures often present atypically with symptoms such as confusion, subtle automatisms, or behavioral changes, leading to frequent misdiagnoses, such as transient ischemic attacks or dementia [15,16]. Furthermore, older adults often experience prolonged postictal states, complicating timely recognition and management [15]. Outcomes are closely tied to the underlying etiology; for instance, cerebrovascular causes are associated with significantly higher short-term mortality compared to metabolic triggers [14,16]. Effective management involves the prompt identification of the precipitating cause and tailored therapeutic strategies that account for the unique pharmacokinetic challenges and comorbidities in this demographic [15,16].

A growing body of evidence underscores the intricate bidirectional relationship between epilepsy and dementia, with Alzheimer’s disease (AD) being the most studied subtype. Epilepsy significantly increases the risk of developing dementia, with hazard ratios indicating a two-to threefold greater likelihood than that in individuals without epilepsy [17,18]. This heightened risk is attributed to overlapping pathological mechanisms such as temporal lobe atrophy, amyloid beta accumulation, tau protein hyperphosphorylation, and chronic neuroinflammation, all of which contribute to neuronal hyperexcitability and subsequent cognitive decline [18,19]. Notably, these shared mechanisms create a feedback loop in which epileptic activity exacerbates dementia pathology, further accelerating memory loss and functional impairment [18].

Conversely, dementia, particularly early-onset AD, predisposes patients to an increased likelihood of seizures and epilepsy. Studies suggest that epileptic activity often manifests in the early or even preclinical stages of AD, potentially serving as a biomarker for the disease [19]. This activity is frequently subclinical and underdiagnosed due to its nonmotor presentation, leading to delays in treatment and further cognitive deterioration. Late-onset epilepsy in older adults is also strongly linked to neurodegenerative processes, with AD patients being particularly susceptible due to disrupted neural networks and impaired inhibitory interneuron function [19].

In older patients, poststroke seizures (PSSs) occurring beyond the acute phase, often referred to as late seizures, pose significant challenges. Late seizures, defined as those occurring more than seven days after the initial stroke event, frequently result from chronic changes such as gliotic scarring, neuroinflammation, and synaptic remodeling that predispose neurons to hyperexcitability [20,21]. These seizures, which are often a precursor to poststroke epilepsy, have a higher incidence in older adults due to increased cortical involvement and higher rates of hemorrhagic transformation in this population [20,21]. Studies indicate that older age, while generally associated with a reduced seizure threshold, also correlates with worse outcomes, including increased disability, recurrent strokes, and heightened mortality [22,23]. Effective management of late seizures in elderly patients is complicated by comorbidities and potential drug interactions, necessitating individualized treatment plans. Antiseizure medications with favorable tolerability profiles and minimal interactions are preferred in this demographic [20,21,24].

A review of five population-based studies on mortality in developed countries summarized that individuals with epilepsy face a 1.6 to 3.0 times higher mortality rate compared to the general population. Remote symptomatic epilepsy has standardized mortality ratios (SMRs) ranging from 2.2 to 6.5. The highest SMRs are observed in children due to the low mortality rates in the reference population, while absolute excess mortality peaks in the elderly, particularly those over 75 years of age [25,26,27,28,29,30,31]. Incident epilepsy is associated with higher mortality when diagnosed after another neurological condition, with mortality increasing alongside comorbid burden [32]. Status epilepticus is an independent predictor of death among the elderly [13]. Our study extends these findings, indicating that age itself may act as an independent predictor of mortality, even after adjusting for confounders such as ischemic stroke, nontraumatic intracerebral hemorrhage, traumatic brain injury (TBI), dementia, brain neoplasms, and status epilepticus.

In our retrospective cohort study, 59% of patients hospitalized with seizures were aged 50 or older. Our findings indicate that, after adjusting for potential confounders, patients aged 50 and older were twice as likely to die during hospitalization, suggesting age as an independent predictor of mortality. Additionally, older patients experienced significantly longer hospital stays and higher hospitalization costs than younger patients, highlighting the broader healthcare burden associated with this age group. These results emphasize the need for age-specific strategies in managing older patients with seizures in hospital settings to improve outcomes and optimize resource utilization.

Our findings are consistent with prior studies showing that older adults with epilepsy often experience worse outcomes due to multiple risk factors, including neurodegenerative and cerebrovascular changes. The age-related synaptic changes that disrupt brain connectivity and decrease the size of the hippocampi are potential processes that increase the likelihood of seizure activity [8]. Polypharmacy and the altered pharmacokinetics in older adults, with increased risk of drug toxicity and interactions, are some factors that may contribute to the prolonged hospital stays and higher mortality rates observed in this population [33]. Socioeconomic implications complicate epilepsy management in older adults. Elderly patients, those over 65 years of age, are known to incur increased hospitalization expenses. The higher costs in elderly patients are attributed to a greater prevalence of comorbidities and complications, which often require more intensive and resource-demanding treatments [34].

Older adults with epilepsy often experience diminished social support and heightened isolation, which can delay diagnosis and worsen outcomes. A longitudinal study highlighted that affectionate support remains stable over time, but opportunities for positive social interaction are limited, often exacerbated by stigma that leads to self-isolation and reduced reliance on broader community networks [35]. Peer support networks, providing both emotional and instrumental support, have shown significant potential in reducing social isolation and enhancing confidence in healthcare management through shared experiences and practical advice. However, barriers such as limited access to Internet-based peer networks, transportation challenges, and regional disparities in professional support disproportionately affect this population [36]. Addressing these challenges through expanded peer-based interventions, stigma reduction efforts, and improved access to healthcare and community resources can play a critical role in improving outcomes and reducing the burden of epilepsy in older adults.

Psychiatric comorbidities, including depression and anxiety, are common in older adults with epilepsy, significantly impacting their quality of life and seizure outcomes. They exhibit higher rates of depressive symptoms and anxiety compared to peers without epilepsy. Additionally, sleep disturbances, such as increased somnolence and poor sleep quality, are prevalent in this population and can exacerbate health outcomes. Addressing these issues through integrated care can enhance both seizure management and overall well-being [37].

The management of seizures in older adults presents unique challenges, as highlighted by this study and supported by the recent literature. Older adults hospitalized with seizures face worse outcomes than younger cohorts, including higher in-hospital mortality, prolonged hospital stays, and increased healthcare costs. These outcomes reflect the interplay of age-related vulnerabilities, including physiological and structural brain changes, and seizure-specific challenges, as well as comorbidities like cerebrovascular disease, dementia, and metabolic disorders, which often compound the complexity of managing older patients with seizures [3,14,16].

Seizures in the elderly are often secondary to structural or metabolic causes, such as stroke or neurodegenerative disorders. Stroke is a major contributor, with seizures occurring both acutely and as late-onset events. The bidirectional relationship between seizures and cerebrovascular disease necessitates careful evaluation for underlying vasculopathies and proactive risk factor management [15,16]. Poststroke seizures often require nuanced differentiation from recurrent strokes or other neurologic conditions, a process complicated by the prolonged postictal states seen in this population [16,18].

From a pharmacological perspective, antiseizure medications (ASMs) must be selected with care to minimize adverse effects, drug–drug interactions, and potential cognitive impairment. Levetiracetam and lamotrigine remain preferred options due to their efficacy and tolerability profiles, while newer-generation ASMs are being explored for their potential benefits in this demographic [17,19,20]. However, evidence from large, randomized controlled trials remains limited, underscoring the need for further research into optimal dosing and long-term outcomes in older adults [19,38].

The psychosocial implications of epilepsy in older adults also warrant attention. Fear of seizures, loss of autonomy, and social isolation often exacerbate the disease burden. Multidisciplinary care models, integrating geriatricians, neurologists, and community resources, are crucial for addressing these challenges. Evidence suggests that a tailored, patient-centered approach can significantly improve quality of life and adherence to treatment regimens [16,20].

The emerging data highlight the importance of non-pharmacological strategies, including cognitive-behavioral therapy and physical rehabilitation, to address the broader impact of seizures on mental health and physical function. These strategies not only complement traditional pharmacological treatments but also address the unique challenges faced by older adults, such as maintaining mobility and independence. Furthermore, addressing comorbid conditions such as osteoporosis, depression, and dementia is integral to optimizing patient outcomes [20,39].

In summary, a multidisciplinary approach, including individualized antiseizure medication selection, close monitoring for polypharmacy risks, and addressing comorbid conditions such as cerebrovascular disease and dementia, is essential for improving outcomes. Non-pharmacologic interventions, including cognitive and physical rehabilitation, caregiver education, and social support initiatives, may also help mitigate seizure burden and improve quality of life in older adults. These strategies, alongside careful medication management, can help reduce hospitalization risks and optimize long-term seizure control in this vulnerable population.

Looking forward, future research should focus on the development of age-specific treatment protocols that account for the unique pharmacokinetic and pharmacodynamic profiles of older adults. Large, randomized controlled trials are needed to evaluate the efficacy, tolerability, and long-term safety of newer ASMs specifically in older populations. Studies should also explore the potential of personalized medicine, including genetic and biomarker-driven approaches, to tailor therapies based on individual patient characteristics and comorbidities.

In addition to pharmacologic treatments, there is a need to investigate the role of adjunctive therapies, such as neuromodulation techniques, dietary interventions, and cognitive rehabilitation, in improving seizure control and quality of life. Given the significant psychosocial burden of epilepsy in older adults, future research should also examine strategies to enhance social support, reduce stigma, and integrate mental health services into epilepsy care.

Finally, population-based studies are essential to better understand healthcare disparities, barriers to specialized care, and the economic impact of epilepsy in this demographic. These findings could inform public health strategies aimed at improving access to care, optimizing resource allocation, and ultimately reducing the burden of epilepsy on older adults and their families.

This study has several limitations. First, as it utilizes the National Inpatient Sample, which includes discharge-level variables coded at the administrative level, it assumes accurate coding. The possibility of diagnosis misclassification cannot be entirely excluded. The National Inpatient Sample (NIS) represents approximately 20% of all hospital discharges and uses stratified sampling and weighting techniques to provide national estimates. However, as it is a sample rather than a census, certain nuances in patient or hospital-level characteristics may not be fully captured. This limitation may restrict the generalizability of our findings to the broader U.S. inpatient population. Furthermore, the database lacks information on medications, test results, and imaging studies, limiting our ability to assess their associations with patient outcomes. Lastly, this is a retrospective study, which limits the capacity to infer causality.

## 5. Conclusions

Our study highlights the significant impact of age on patient characteristics and outcomes in seizure-related hospitalizations using a nationally representative dataset. Older adults, representing the majority of seizure-related hospitalizations, exhibited distinct demographic patterns, a greater burden of comorbidities, and worse outcomes compared with younger patients. These findings underscore age as an independent predictor of in-hospital mortality, longer hospital stays, and higher healthcare costs, even after adjusting for potential confounders.

This study also sheds light on the unique challenges faced by older adults with seizures, including the influence of neurodegenerative changes, cerebrovascular disease, and polypharmacy, which contribute to their heightened vulnerability. Psychosocial factors, such as limited social support and increased stigma, further exacerbate the disease burden in this demographic. Additionally, pharmacologic and non-pharmacologic strategies, as well as integrated care models, were identified as critical components in improving outcomes for older patients.

Future research should prioritize the development of age-specific treatment protocols that address the unique vulnerabilities of older adults, while exploring innovative approaches such as neuromodulation and personalized medicine. Addressing healthcare disparities and barriers to care through public health initiatives will be essential in reducing the economic and clinical burden associated with seizures in the aging population.

By emphasizing tailored management strategies and a multidisciplinary approach, including both medical and psychosocial interventions, this study provides a foundation for improving outcomes and optimizing resource utilization for older adults hospitalized with seizures, while also informing future research priorities in this area.

## Figures and Tables

**Table 1:** Patient demographics and hospital characteristics.

	Age ≥ 18–49	Age 50 and Older	*p*-Value
Female (%)	40,000 (46)	61,000 (49)	<0.01
Mean age (95% conf. interval)	33.62699 (33.451 33.80298)	67.34032 (67.17088 67.50975)	<0.01
Race/ethnicity (%)			<0.01
White	46,000 (54.26)	74,000 (62)	
Black	20,000 (23.69)	30,000 (24.58)	
Hispanic	13,000 (15.09)	11,000 (8.97)	
Asian or Pacific Islander	1730 (2.05)	1825 (1.51)	
Native American	765 (0.9)	755 (0.63)	
Other	3385 (4.0)	3310 (2.74)	
Median annual income in patient’s zip code, USD, No. (%)			<0.01
USD 1–51,999	28,000 (33.44)	41,000 (33.73)	
USD 52,000–65,999	21,000 (24.63)	30,000 (24.49)	
USD 66,000–87,999	20,000 (23.52)	27,000 (22.16)	
USD 88,000 or more	16,000 (18.41)	24,000 (19.62)	
Insurance type, No. (%)			<0.01
Medicare	13,000 (15.53)	79,000 (66.03)	
Medicaid	36,000 (42.74)	18,000 (15.2)	
Private insurance, including Health Maintenance Organizations (HMOs)	27,000 (32.47)	19,000 (15.62)	
Self-pay	7720 (9.26)	3765 (3.15)	
Hospital region, No. (%)			<0.01
Northeast	18,000 (20.64)	26,000 (20.66)	
Midwest	19,000 (22.04)	27,000 (21.43)	
South	34,000 (38.46)	51,000 (41.14)	
West	16,000 (18.86)	21,000 (16.78)	
Urban location (%)	84,000 (96.35)	120,000 (94.4)	<0.01
Teaching hospital	74,000 (84.41)	98,000 (78.93)	<0.01

**Table 2:** Relevant clinical characteristics.

	Age ≥ 18–49	Age 50 and Older	*p*-Value
Patients with a principal diagnosis of status epilepticus (% of total seizure admission)	14,000 (15.91)	21,000 (16.64)	0.07
Comorbidities			
Charlson Comorbidity Index score, No. (%)			<0.01
0	58,000 (66.51)	29,000 (23.76)	
1	16,000 (18.42)	28,000 (22.93)	
2	7055 (8.09)	23,000 (18.66)	
≥3	6080 (6.97)	43,000 (34.65)	
Ischemic stroke (%)	380 (0.44)	2635 (2.13)	<0.01
Nontraumatic intracerebral hemorrhage (%)	555 (0.64)	1970 (1.59)	<0.01
TBI (%)	1685 (1.93)	2455 (1.98)	0.73
Dementia (%)	415 (0.48)	22,000 (17.44)	<0.01
Brain neoplasms, both primary and metastatic (%)	1370 (1.57)	4845 (3.91)	<0.01

**Table 3:** Primary outcome.

Outcome	Age Group	Odds Ratio (OR)	95% Confidence Interval (CI)	*p*-Value
Mortality (unadjusted)	50 and older	3.73	2.95–4.73	<0.01
Mortality (adjusted)	50 and older	2.17	1.61–2.92	<0.01

**Table 4:** Secondary outcomes.

Adjusted Secondary Outcomes	Value	95% Confidence Interval (CI)	*p*-Value
Mean length of stay (50 and older)	0.7 days longer	0.54–0.92 days	<0.01
Total hospital charges (50 and older)	USD 4322 higher	USD 1914–6731	<0.01

## Data Availability

The data presented in this study are available on request from the corresponding author. The National Inpatient Sample (NIS) database, curated by the Agency for Healthcare Research and Quality (AHRQ), requires users to complete mandatory training and purchase access. Details on accessing the data can be found on the HCUP-NIS Database Documentation website.

## Supplementary Materials

The following supporting information can be downloaded at: https://www.mdpi.com/article/10.3390/neurolint17030039/s1, Table S1: ICD-10 codes.
