# Supplementary material for: The Impact of Age on Outcomes in Seizure Hospitalizations—Analysis of a National Sample
Source: Neurol Int. 2025 Mar 4;17(3):39. doi: 10.3390/neurolint17030039 (PMC11944413; doi:10.3390/neurolint17030039)
Supplement: Supplementary file 1 [file neurolint-17-00039-s001.zip › neurolint-3417421-supplementary.pdf]

Table S1

**Diagnosis**	**ICD 10 codes**
Seizures	G40x, G41x and R56x
Status epilepticus	G41.0, G41.1, G41.2, G41.8, G41.9, G40.A01, G40.A11, G40.B01, G40.B11, G40.401, G40.301, G40.311, G40.411, G40.201, G40.211, G40.001, G40.101, G40.011, G40.111, G40.501, G40.801, G40.803, G40.811, G40.813, G40.823, G40.821 G40.833, G40.901, G40.911
Ischemic stroke	I63x
Non traumatic ICH	I60x, I61x, I62x
TBI	S06x
Dementia	G30x, F01x, F03x, G31.01, G31.09, G31.83
Bran neoplasm	C710, C711, C712, C713, C714, C715, C716, C717, C718, C719, D330, D331, D332, D333, D334, D337, D339, D430, D431, D432, D433, D434, D438, D439, C7931, C7932

X = all the subcodes following the trunk
